# Beneficial and Impeding Factors for the Implementation of Health-Promoting Lifestyle Interventions—A Gender-Specific Focus Group Study

**DOI:** 10.3390/ijerph20043520

**Published:** 2023-02-16

**Authors:** Felix G. Wittmann, Andrea Zülke, Adrian Schultz, Mandy Claus, Susanne Röhr, Melanie Luppa, Steffi G. Riedel-Heller

**Affiliations:** 1 Institute of Social Medicine, Occupational Health and Public Health (ISAP), Faculty of Medicine, University of Leipzig, Philipp-Rosenthal-Str. 55, 04103 Leipzig, Germany; 2 Health and Ageing Research Team (HART), School of Psychology, Manawatū Campus, Massey University, Palmerston North 4474, New Zealand

**Keywords:** older people, gender-specific perspectives, lifestyle change, prevention, intervention, dementia

## Abstract

(1) Background: The prevalence of dementia increases and so does the number of interventions that address modifiable risk factors for dementia. Recent evidence suggests that there are gender differences in the prevalence of those lifestyle factors as well as in the effectiveness of interventions. This study aims to identify differences in factors that benefit or hinder the effectiveness of interventions since a target group’s perspective gets more relevant. (2) Methods: Two focus groups, a female (*n =* 11) and a male (*n =* 8) group, were interviewed, audio recorded and transcribed. Qualitative analyses were performed and main- and subcategories were identified. (3) Results: Main differences were observed including aspects of lifestyle changes (e.g., respective diet and importance of an active lifestyle) and gender-typical behavior and perception by relevant healthcare actors. (4) Conclusions: Identified differences might help to address and raise the efficiency of lifestyle interventions. Further, the importance of social aspects and retirement as an auspicious moment to start interventions were identified as relevant by study participants.

## 1. Introduction

Worldwide, the prevalence of people living with dementia is estimated at 55 million people [1]. The number is expected to triple up to 139 million in 2050 [1,2]. Available treatments are only able to improve cognitive function over a limited period of time and are not yet curative [3]. However, 40% of the risk of dementia seems to be amenable and able to change by modification of risk and protective factors. Livingston and colleagues identified twelve such potentially modifiable risk factors. These are, among others, low education and obesity in midlife but also smoking, excessive alcohol consumption, physical inactivity or depression and social isolation [4]. This is why there is increasing interest in lifestyle interventions to modify these risk factors [5]. Furthermore, addressing these risk factors through interventions as a form of prevention is an essential part of dementia risk reduction defined by the World Health Organization (WHO) in their global action plan on the public health response to dementia [6].

The prevalence of dementia differs between genders [7,8] as well as risk factors. While there are non-modifiable risk factors as the genotype Apolipoprotein E, there is growing evidence of differences in modifiable risk factors for dementia in men and women [9]. Therefore, risk factors may differ in one or both the frequency or the strength of the effect, or are even restricted to just one sex [10]. A classic example is, as mentioned above, low education. Although it has the same effect on dementia risk for both men and women, it is historically more common in women [9] due to unequal opportunities for schooling in older cohorts. Other risk factors that enhance the risk for women are history of stroke, little physical activity and hopelessness in late life, while for men history of stroke and more severe insomnia in late life are of greater relevance [8].

Within the last few years, multi-domain lifestyle interventions have been investigated in over 40 countries. As the first randomized controlled trial (RCT) targeting cognitive function in older adults at increased risk for dementia, the Finnish Geriatric Intervention Study to Prevent Cognitive Impairment and Disability (FINGER) reported positive intervention effects on global cognition, domain-specific cognitive function and further physical functioning [11]. Although the effects reported so far are rather small, there are efforts to apply a global network, the World-Wide Fingers network. It pursues the goal of optimizing the FINGER-approach and adjusting it to different regional, thus economic and cultural environments [12,13]. Additionally, growing evidence of secondary data analysis provides new knowledge about intervention-related factors and effectiveness, e.g., in several subgroups of respective trials [11].

A recently published review by Zülke and colleagues on the gender-specific effectiveness of lifestyle interventions on cognitive functions did not find significant effects of lifestyle interventions on global cognition in either male or female samples. Nevertheless, when stratifying by gender the included studies indicated a positive effect of lifestyle on cognitive functions only in women. However, there is a lack of studies for male participants or trials reporting gender-stratified results [14]. Reviewing physical activity interventions, Barha and colleagues reported higher benefits for women than men in executive processes and greater benefits of aerobic training than other exercises [15]. Finally, Sindi and colleagues did not report any significant differences between men and women for the FINGER-study. However, gender-specific analysis mentions the relevance of differences in vascular, lifestyle and psychosocial factors [8].

Evidence suggests that gender plays a crucial role in the prevalence and in modifiable risk factors in dementia. It might thus be relevant to further take gender and respective differences into account to increase the effectiveness of future lifestyle interventions. Previous studies show a slight advantage of interventions for women, while men are often underrepresented. In a paper about current developments, Röhr and colleagues filtered out first factors that might facilitate but also hinder intervention adherence [11]. The current state of research forms a stable and assertive basis for being able to align even more targeted and efficient lifestyle interventions in the future. However, to our knowledge, little is known about respective factors differing by gender. This study aims to contribute to this growing area of research by identifying factors that benefit or hinder the implementation of lifestyle interventions to modify risk factors for cognitive decline in older age as identified by older men and women, respectively. Focus group interviews separated by men and women might give information about how the uptake, the adherence and the effectiveness of interventions might be enhanced. Further, results might inform about previous problems, such as low participation in men, and might highlight the needs and requests of older people for worthwhile lifestyle changes.

## 2. Materials and Methods

### 2.1. Study Sample

Potential study participants were contacted using a pool of previous participants from other studies who had agreed to be contacted again. Participants were recruited between March and April 2022. Inclusion criteria were experiences in lifestyle changes and an age of 60 years and above. The change could either have already been experienced or be currently ongoing.

### 2.2. Study Design

Two gender-homogeneous focus groups were interviewed by a trained member of the research group. We used focus group interviews since they benefit from group dynamics and social interaction and therefore often provide deeper and richer data than individual interviews [16]. Furthermore, the focus should be on the perspective of the target groups, which is why group discussions were used. The study was conducted in accordance with the standards for reporting qualitative research recommendations (SRQR) [17].

The interviews with the focus groups both followed a semi-structured interview approach. First, questions about the importance and aspects of lifestyle changes were asked. Participants were then asked about bigger changes they already had implemented. Causes for these changes and appropriate handling were discussed. Then, the groups were asked about how they fared and which affect different circumstances had. Participants were finally asked about different factors that either facilitate or hinder appropriate changes. The focus was expanded to the differences between men and women respective of these factors.

The interviews were audio-recorded and in the next step transcribed by an external company.

Participants’ sociodemographic characteristics were assessed by a paper questionnaire. Six questions regarding gender, age, familiar situation, graduation, vocational training and employment status were surveyed.

### 2.3. Analysis

The transcripts were analyzed and revised by two researchers (F.W, A.S) independently according to Mayring and Fenzl [18]. Category formation followed both deductive (based on the interview guide) and inductive (based on the answers) approaches. After a first pre-encoding performed individually by each research assistant, categories were reviewed and aligned before they were recorded separately again. The final category system and respective example quotations were finally defined and coded in a last group discussion. Analysis was carried out using the software MaxQDA 2018 (VERBI Software, Berlin, Germany).

## 3. Results

### 3.1. Sample Characteristics of the Focus Group Participants

We recruited 19 persons of which 8 were men and 11 were women between 60–80 years of age, with an average age of 66.3 ± 5.5 years. Participant characteristics are shown in Table 1.

### 3.2. Themes and Subthemes Identified

We identified six themes each including three to six subthemes. Table 2 shows the frequency of quotes per subcategory overall and separated by gender.

The six themes identified were aspects and triggers of lifestyle change, differences between men and women, resources and barriers as well as needs and requests. It can be seen from the data in Table 2 that women more often mentioned aspects of lifestyle change, differences, especially characteristic traits and barriers. Further, besides diseases, retirement was mentioned comparatively often but in both a negative and a positive way. The subcategories are elaborated including example quotations in Table 3.

#### 3.2.1. Aspects of Lifestyle Change

Aspects of lifestyle change mentioned by the male and female focus group were quite equal in psychosocial factors and activity, whereby activity contained mostly sport and was also brought up as a remedy against pain:

*“And I was at the orthopedists again recently. He said: “I don’t need shots. I don’t need it. It all hurts me. But I’ll always get it when I am exercising now.” Early in the morning a quarter of an hour, and then from the beginning. With my husband. He is also doing this. And then we ride our bikes or go for a walk, and then our exercises.”*

(female participant)

Statements about healthy diet were more common in the male group. The growing importance of a healthy diet was attributed to retirement age and diseases linked to aging.

Positive aspects of social contacts were highlighted in both the female and the male group. Especially family, but also acquaintances, were mentioned. Mutual help and understanding were seen as important as well as engaging oneself actively:

*“And it is also important that you find ways to communicate. A hiking group and other such things. And then I started to do something for that.”*

(female participant)

Men further accentuated the common household and domestic peace and broached the reciprocal effect of health and social environment:

*“Participating in social life, also in cultural life. Yesterday evening we went to the theatre, for example. We were part of a drama performance. That is part of it all. And this is not only true when I am healthy enough that I can do this, that I can go to the next village, get into it and hold it out for one and a half hours. And my group of friends is also important for staying healthy.”*

(male participant)

An aspect mentioned only by women was an active lifestyle. Statements towards an active lifestyle contained an active way of life or self-determination, whereby being (still) able or allowed to work was considered an important factor with respective benefits:

*“For myself, a healthy lifestyle only applies to me personally today—that I still have my job. That I can still work, because that is where I get my energy. And as she [other participant] has said, it is thankfulness that comes across, acceptance, friendliness. It’s clear, and you can take whoever you want. It doesn’t matter. There are positive and negative experiences everywhere, but that is my source of energy. And of course the home and the yard. A few weeks ago we got some chickens. That is also quality of life for us.”*

(female participant)

Additionally, quitting smoking came up as an aspect of a lifestyle change in the male group.

#### 3.2.2. Triggers and Causes of Lifestyle Change

Triggers of recent lifestyle changes were categorised into diseases, psychosocial factors and retirement. While the extent of diseases mentioned as triggers was quite equal in general, men more often stressed diabetes as a specific disease:

*“I had to get a new doctor. The doctor told me I have diabetes. And that I really had to lose weight.”*

(male participant)

Statements about psychosocial factors were more often made by women than men. While women named grandchildren or the spouse as the source, men commonly named psychotherapy or group therapy as triggers of lifestyle changes.

Retirement was seen from both a positive and a negative perspective. Some women were happy about their husbands retiring because they felt it was a relief in everyday life. Men, however, mentioned the relief of being able to calm down when retiring. In addition, retirement was seen as a challenge, since both, but mainly women mentioned having fallen into an emotional chasm and having had the challenge to build up new daily structures.

#### 3.2.3. Differences between Men and Women

The main category differences between men and women were subdivided into three subcategories: general differences, characteristics and behavioral differences. While women mostly stressed the different interests between men and women, they also mentioned the importance of mutual help:

*“Helping one another is important.”*

(female participant)

Also, traditional social gender roles were mentioned regarding differences:

*“(…) But since she [other participant] just said about what it has to do with that: you are a man, you have to be strong.”*

(female participant)

Men, however, in addition to differences, see type-related and psychological factors as important factors that transcend gender differences. Nevertheless, they also see the axis between emotional and rational orientation as clearly attributed:

*“I am a relatively strong objective person. And my wife is, in contrast, much more emotionally oriented. The world of emotions and the objective world… Objectively I can be very direct in arguing and fighting with you. If you are a sensitive person, an objective matter that concerns you can release so much frustration, that it (…).”*

(male participant)

Supposedly typical character traits were mentioned more often by women than men. A common statement of the female group was that men are in general less emotional and open and do not seek external help when confronted with problems:

*“That is why they deal with a lot of things inside themselves. They don’t talk about their problems.”*

(female participant)

Regarding behavioral differences, the views within the women’s group were inconsistent. Regarding seeking advice or getting help, for example by seeing a practitioner, women said both that men hesitate more and need some time to finally get help from a doctor, or that they are more rigorous and get help from a medical professional directly:

*“Or it takes longer before they go for help … and then, well, when it doesn’t work at all.”*

(female participant, in comparison to the quote in Table 3)

Men explicitly only talked about their wives when talking about women, and instead were quite in agreement about themselves being able to balance reasons better than their female partner:

*“I set myself a goal. I want to do it that way. My wife starts to think. If I do it that way, that’s the way it is. If I do it that way, that can be the result. And she thinks a lot about the whole matter. It’s simple, and she doubts a lot, whether it is right or wrong. That doesn’t happen to me. I don’t have doubts. I want to do it now. And then I’ll see what happens. With her it is a little different.”*

(male participant)

#### 3.2.4. Resources and Support

The first subcategory of resources was identified as social environment, which was addressed to a greater extent by women. For women, family and acquaintances were mentioned as positive resources for lifestyle changes. A point that came up several times was that in friendships and with acquaintances people sometimes come up with arguments that may not suit oneself but are nevertheless important to consider:

*“I was glad when I had friends, and then you sometimes have to accept some things, that they sometimes say things you don’t want to hear.”*

(female participant)

Men more often mentioned their children and their spouse as social resources. Important factors here were social pressure:

*“Pressure from the immediate environment, whenever pressure is put on the family or the children, like, the way you are doing that is not allowed.”*

(male participant)

Another important factor was accolade:

*“Yes, and then the family accepted it. I stopped smoking. And here we are with praise and condemnation again.”*

(male participant)

Besides the social environment, the healthcare system was mentioned as in some form supportive, but with different aspects highlighted in men and women. Men in general named their general practitioner as supportive, especially regarding information about preventive check-ups. Women stressed the beneficial effects of group therapy, self-help groups or group sessions on respective pain. However, also other ways of support from the health sector were mentioned:

*“That was my psychotherapist. There was my coach in the fitness center. There is the orthopedist and my psychotherapist. They helped me get it back together over the years.”*

(female participant)

The third subcategory of resources was psychosocial factors. Women named mainly positive attitude, self-acceptance and mutual understanding, while for men setting goals for themselves and reaching them was important.

Social factors were the last subcategory of resources. While this subcategory did not come up to a bigger extent in the group of women, men made more statements about it. In general, community and joint experiences were mentioned. An example is:

*“Exactly. For organizations in every direction, whether sport clubs, bowling clubs. Bowling is sports. Whether hiking clubs or craft evenings, whether afternoons for senior citizens, or get togethers for senior citizens, sport groups for the retired, those are all things that invite people into social groups.”*

(male participant)

#### 3.2.5. Barriers

Barriers to lifestyle changes were divided into four subcategories: social conditions, personal factors, healthcare system and infrastructure.

Social conditions were held as a broader subcategory and the discussions about problems regarding lifestyle changes and respective social conditions developed in different directions. Women in general mentioned missing centres of cultural and social life or specific places in the city to go to get help several times. Furthermore, financial aspects were stressed a lot:

*“If you go somewhere, you need money. If you have none, you can’t go or drive. This is all a questions of finances or what is available.”*

(female participant)

In the male group, the main problems addressed concerned societal life and the difficulty of getting along with alienation from the social or residential environment. These problems were linked to employment and persisted even after retirement.

Personal factors were addressed in both groups but to a lesser extent. Women mentioned less physical and mental strength, in part due to the loss of the partner as a turning point in life:

*“In addition, the big change came for me two, really? Two years ago. And it was then when I had a strike of fate. It was really a shock. And life suddenly changes, for everything. My husband suddenly died. And then my nutrition changed. We have a house with 1000 square meters that has to be taken care of and kept up to date. That’s the way it is…”*

(female participant)

Men mentioned demands from the environment but also on themselves as hindering:

*“My burdens come from my demands on myself”*

(male participant)

Problems regarding the healthcare system were mentioned in both groups with similar arguments and to a bigger extent by women. A mutual persuasion was that the healthcare system, insurances, but also and especially practitioners prioritize financial interests over patients. Women furthermore mentioned a lack of commitment, and the quality of their general practitioners was criticized:

*“Nowadays you go to the doctor and you know what to say to the doctor. And you tell the doctor in detail what you want to have and what you want (agreement). You are hardly examined anymore. Usually the symptom is treated and not the cause. That is why you have to go to the doctor already convinced and say: “We have to do this, and this, and that.” Or, since I am very impulsive and read a lot, I will also usually be treated according to the way I want.”*

(female participant)

Infrastructure as a barrier was mentioned only in the group with female participants. Especially the urban-rural contrast turned out to be relevant. Two aspects, in particular, should be mentioned here. First, social aspects are seen negatively in the urban contexts, as cities are perceived as more anonymous:

*“But you have your social aspects when getting groceries. You see a familiar face and can speak with the person. In a city it is all anonymous.”*

(female participant)

Second, cities are seen as an advantage, and places that provide opportunities for social encounters are named here since they are only to be found in cities.

#### 3.2.6. Needs and Requests

Needs and Requests were not mentioned to a big amount, but three subcategories could be identified: societal, healthcare and cultural aspects.

Statements that were mentioned within the societal subcategory are linked to aspects stressed out in the barriers category. Concrete places to go to get help are desired here as well as more social work from the community or the local authority:

*“A social worker in the city. Like in a hospital. That kind of thing, like a place to get help in the city.”*

(female participant)

A more concrete example is mentioned the following:

*“I have a friend in assisted living, and that is where it is organized. Next door is the nursing home. And in the cafeteria there are things for elderly people to eat. You can also come from other places, take part in game days, play games. They put on a Christmas party and all kinds of things with cooking out and with a bazaar. And I think it is nice that it is all accessible for the public, and for elderly people and what they do. You can take advantage of such things. You don’t have to live there—you can go along with someone.”*

(female participant)

Statements on the healthcare system are also linked to barriers to lifestyle change. Little of this was mentioned, but it was coherent: more time and more information are desired from general practitioners.

Finally, cultural aspects came up in the female group. There are complaints about a lack of supply.

## 4. Discussion

Our study is the first to use a qualitative approach to examine factors that, separated by gender, impact the implementation and effectiveness of lifestyle changes in older age. In this way, we contribute findings to the current state of research, which should make it possible to carry out lifestyle interventions in a more targeted and efficient manner.

A very important aspect that was mentioned in several ways was the social environment. While it was mentioned in an equal amount as an aspect of lifestyle change in form of a supportive factor, women stressed social aspects to a greater extent as a resource. This ties in with Tomioka and colleagues who report more participation in social activities and an association with a lower decline in cognitive function only in women, not in men [20]. These results support evidence from previous research on the social environment and engagement as important aspects of aging [21,22,23], but especially, what we want to mention here, as an aspect of lifestyle changes. Enhancement of social activity was targeted only in a limited number of trials so far [14]. It can thus be suggested, that social aspects such as environment, activity and engagement could be crucial adjuncts in interventions to enforce a change in lifestyles as they are a key modifiable factor regarding cognitive performance [24]. Social activities are also often linked to other resources, e.g., combined with physical activity [25]. Similar arguments were also mentioned in our results by participants who linked social activities with different domains. These are, e.g., support, social pressure or last but not least social demands enforced by norms. In a quite new conceptual framework, Vernooij-Dassen and colleagues mention the factors of structure and function (e.g., exchanging support) and appraisal of the social environment as main aspects of social health in relation to dementia and also emphasize the relevance for interventions [26].

Some differences between men and women could further be observed regarding attitudes toward the healthcare system. First, different aspects were mentioned regarding the healthcare system as supportive or as seen as a resource. Men stressed the supportive behavior of their practitioners, for example, regarding information received about preventive medical check-ups. Women, however, did not mention their general practitioner as a resource. Instead, psychotherapy and group sessions (e.g., self-help group or group therapy for persons with chronic pain) were brought up in the female group. While problems regarding the healthcare system were quite equally mentioned, another difference could be made out by analyzing barriers that the participants named in the interviews. Both groups complained about the fact that, according to their experience and opinion, financial aspects play far too big a role in providing care and treating patients appropriately. Nevertheless, the female group came up with more barriers than men. They further mentioned a lack of support by the healthcare system, the feeling of not being taken seriously or the declining quality of practitioners. These results support an argument made by Mauvais-Jarvis and colleagues, that gender might matter in both patients and doctors behavior. According to Mauvais-Jarvis and colleagues, gender roles depict social and thus behavioral norms and may thereby influence the access to healthcare systems, help-seeking behavior and the use of healthcare systems [27]. Further, Sieverding and colleagues report gender stereotypes within the doctor–patient communication. An interesting example of the study is that women are more likely to get a psychological diagnosis, men a somatic diagnosis for the same symptoms [28].

When discussing differences in characteristics or behavior between men and women, both groups mostly agreed about men being more pragmatic and rational, dealing with problems by themselves, while women were seen as more affective, e.g., by trying to amplify efforts of positive emotions and being more open to getting help was an argument that was very strongly emphasized in both groups. This might be an addressable point for further interventions relevant to the point that men are often underrepresented in lifestyle interventions. Pagoto and colleagues discussed possible reasons for this in relation to obesity interventions. Possible reasons, the authors argue, include social norms about healthy lifestyles that are more common in women, for example, social pressure. Further, the different interest in seeking outside help is discussed. This is in line with our results, reporting women being more open to getting help. Therefore, they mentioned quite a large amount of positive aspects of group sessions or getting help from therapists, while men were being connected to self-efficacy and dealing on their own. Finally, Pagoto and colleagues report that different representations of men referred to the type of intervention. While the lowest representation of men was observed in group-based interventions, they were represented to a larger amount in self-guided interventions [29]. To raise male participation and further the effectiveness of interventions, gender as a social construct, instead of or in addition to gender only being seen as a biological marker, should be considered in future investigations towards lifestyle interventions. A way of doing so might be different types of interventions, based on different characteristics and behaviors [27].

Further differences could be recognized in aspects of lifestyle. A healthy diet was mentioned more often in the male group, which is contrary to a focus-group study by Schladitz and colleagues [23]. In addition, an active lifestyle was only mentioned in the female group as an aspect of lifestyle change. However, men emphasized that they were happy to be able to pursue their own activities, e.g., gardening, after retirement. This result is also contrary to the findings of Schladitz and colleagues who found men more focused on an active lifestyle or meaningful activities like intellectual activities, while women did not focus on an active lifestyle. Interestingly, in our findings, women mostly referred to their job as an active lifestyle. This highlights another difference in our results—the appraisal of retirement. While women mentioned positive aspects of employment, men talked overwhelmingly positive about their retirement (e.g., to calm down, having time for hobbies, being able to support their wives in domestic work and reported relaxation). Besides diseases, retirement was the trigger mentioned more frequently for a lifestyle change (cf. Table 2). This result reflects those of Motegi and colleagues. They report changes in lifestyle habits after retirement such as a reduction in drinking, an increase in walking and heavy exercises and sleep time [30]. This might be relevant for the timing of the lifestyle change and thus as a moment to implement interventions. In order to increase male participation, the starting point should be taken into consideration, as this was significantly mentioned in the male group.

Finally, a closer look needs to be taken at barriers that participants reported towards lifestyle changes. While aspects of the social and the healthcare system have already been discussed, infrastructure and cultural aspects stand out. Apart from an urban–rural gap, financial aspects are crucial in both; for people living in more rural environments, missing infrastructure makes it difficult to take advantage of offers that are usually only found in cities. The same applies to culture, such as visits to the theatre or social meeting places. Interestingly, these points were only mentioned by female participants. This fact might be explained by education and occupational history differences [14]. The observable gap between gender and socioeconomic status and resources is important and should thus be considered in lifestyle changes and lifestyle interventions since it might affect seizing health-beneficial offers (as far as not included in interventions).

Links between gender differences and lifestyle changes, respective to the implementation and effectiveness of lifestyle interventions need to be examined more closely. Clinical practice nevertheless should regard gender roles for example by implementing different types of interventions due to differences in characteristic traits and behavior or a gap between gender and socioeconomic status and resources.

This study made use of qualitative data. Therefore, we obtained valuable insights into the experiences of older people regarding lifestyle changes. Thanks to the qualitative approach, we were further able to identify factors influencing the successful implementation of lifestyle changes in advance. Nevertheless, this study has several limitations. Since the interviews were held in German, the results are only partly representative due to the non-representation of migrant or minority groups. Further, it can be assumed that the participation is based on an existing interest of the participants in the topic. The male participants were already retired, while some of the female participants were still employed. This is partly reflected in statements about retirement. Although the sample is not homogenous, sample characteristics are still comparable to those of already existing interventions.

## 5. Conclusions

This study set out to analyze factors influencing the implementation of lifestyle changes as perceived by older men and women, respectively. The results of this research show that there are gender differences in both beneficial and hindering factors. Differences were observed in aspects of lifestyle changes, with diet stressed by men and an active lifestyle in general by women. An important difference can be reported referred to the perception of the healthcare system, partly due to traditional gender roles and accompanying behavior and perception. This could be of relevance for addressing and interacting with people relevant to lifestyle interventions. Social aspects as important factors were mentioned as well as aspects of lifestyle change in form of motivation and also as resources towards changes. Since social aspects have been addressed in a far too little manner in recent trials, but are seen as very efficient and important, they should be focused on more strongly. Finally, since retirement was seen as an important trigger for changes and also as a chance for new beginnings, this might be a very fertile moment to implement lifestyle interventions. This new understanding should help to improve the involvement of older people in lifestyle interventions as well as to improve the effectiveness of future interventions.

## Figures and Tables

**Table 1:** Sample characteristics.

Characteristics	Men (*n* = 8)		Women (*n* = 11)	
Age (Mean, (SD))	68.5	(4.8)	64.6	(5.6)
Familiar situation (*n*, %)				
Married	7	87.5	8	72.7
In relationship	-	0	1	9.1
Divorced	1	12.5	1	9.1
Widowed	-	0	1	9.1
Education * (*n*, %)				
Low	1	12.5	1	9.1
Middle	2	25.0	7	63.6
High	5	62.5	3	27.3
Employment status (*n*, %)				
Full-time	-	0	3	27.3
Part-time	-	0	1	9.1
Retired	8	100	7	63.6

Note. * Education: according to CASMIN Classification (Comparative Analysis of Social Mobility in Industrial Nations) [19].

**Table 2:** Frequency of quotes per subcategory overall and separated by gender.

Theme		Subtheme		Overall	Women (*n* = 11)	Men (*n* = 8)
I	Aspects of lifestyle change	i	Activity	16	10	6
		ii	Diet	10	2	8
		iii	Social environment	11	5	6
		iv	Psychosocial factors	11	7	4
		v	Active lifestyle	8	8	-
		vi	Quit smoking	3	-	3
II	Triggers of lifestyle change	i	Diseases	12	5	7
		ii	Psychosocial triggers	6	5	1
		iii	Retirement	13		
			Advantage	8	1	7
			Disadvantage	5	4	1
III	Gender Differences	i	In General	13	6	7
		ii	Characteristic traits	14	12	2
		iii	Behavior	10	3	7
IV	Resources/Support	i	Social environment	17	12	5
		ii	Healthcare system	25	15	10
		iii	Psychological resources	6	4	2
		iv	Social factors	13	5	8
V	Barriers	i	Social conditions	18	13	5
		ii	Personal factors	13	9	4
		iii	Health care	20	16	4
		iv	Infrastructure	5	5	-
VI	Needs/requests	i	Societal	10	7	3
		ii	Health care	7	4	3
		iii	Culture	1	1	-

Note. The coloring of the subcategories corresponds to the frequency of quotes separated by theme, respectively.

**Table 3:** Themes and key quotes identified and separated by gender.

Theme	Subcategory	Quotations
T I: Aspects of lifestyle change	SC i: Activities	(w) Swimming and such things in the meantime. It really helped. Then I bought a bike and rode it and so forth. I went hiking with staffs and…. So a totally different lifestyle has developed.
		(m) The stress as a construction manager, what you have to put up with every day. The guys know it. And somethings you need … Then I started exercising—that was 2002. That helps me free my mind. If I am biking for two hours, then it’s gone. And that is very important. That is how I got into sports again, it’s that simple, because it helped me. That is what I have experienced.
	SC ii: Diet	(w) As I said, nutrition … During that time I lost up to 20 kilos, because you eat differently then. But my awareness of that was different. We ate differently before then [*retirement*] and now. And yeah, it became clear to me: Now you have to stay healthy!
		(m) I said: “So, now we eat differently. Now there are three meals a day: morning, noon, and evening.“ And earlier the evening meal was big, because I only ate sandwiches during the day. But since then I’ve been eating a sandwich in the evening. That’s all. And my wife can fill my plate however she wants, and that’s all. And I notice that. Now for several years I weigh about 70 [*kilograms*] all the time and a little below that.
	SC iii: Social environment	(w) As I said, friendships are really important. I am careful with that. Yes, I had two girlfriends. But something is said which makes you stop and swallow. And then you think about things. I say that then you don’t say anything for about two hours, and then everything is good again. I think that is important.
		(m) For me an extremely important factor is how my wife and I approach each other, and that we don’t only see our own idea as the most important thing. I should also try what she says, even when I disagree with it at the moment. But I also find that one or two days later I have processed what she suggested. And then I try to create a better climate for getting along. Approaching each other, accepting each other with all the mistakes and the nice sides each one has helps a lot to be happy and satisfied and to get rid of stress. And this helps both of us with our health.
	SC iv: Psychosocial factors	(w) Because you still sometimes need the grip, also when you know it. I mean, everything… you read a lot or get informed. And yeah, you can do that, and you have to do it. But when you do it, you sometimes need confirmation. That’s the way it is with me. I need confirmation from her [*psychotherapist*]. That she then once in a while says, “Just do it now! Rest yourself more.” I still can’t do that today.
		(m) How you deal with all of it—and the stress. And then you have to make points of emphasis, if you… and not only take in everything. You have to make points of emphasis, and after some time this has worked well for me.
	SC v: Active lifestyle	(w) But I tried, a little something [*asking for the possibility to engage herself for social activities in the commune*]… So I can get out of my shell. Not always this daily routine, so that something else happens. Good. That is very important, that you don’t become so numb in old age.
	SC vi: Quit smoking	(m) I can say that I could stop smoking from one day to the next. That was in 2010. I was 40 years old and sometimes smoked a lot, and I had a bad conscious for that again and again. That’s all clear. That was a spontaneous sentence that was like flipping a switch. So the electricity source was cut off. And afterwards I had not great difficulties. In social groups where people smoked, sometimes I had also smoked. Oh, okay, now you can have one, but I held out, and it was not a problem at all for me. I had it in my head.
T II: Triggers of lifestyle change	SC i: Diseases	(w) Yes, I had two back operations. I broke my back. I fell down the stairs. But it’s like this: you fight. Your lifestyle changes completely.
		(m) As I said, somewhere a small illness has to appear, so that you will say: now you have to get started or it will go downhill.
	SC ii: Psychosocial triggers	(w) It’s all about trying something new. Try to move on. I got myself a bike last year and I thought, well, now you ride your bike.
		(m) Somehow I learned a lot in the group discussions, and how each person presents himself. Something of that always stays with you. And he [*participant of the group*] said, “Why does he have such things? You can also try that.” That’s the way it is, and it somehow works. Finally all come together and you say… And if you then … If you say about the way things are today: you did it all right. You can say: “Yes, it was good.”
	SC iii: Retirement (+)	(w) And then I was really looking forward to my husband retiring in his early 60 s without any deductions. And that was four years ago. And I have to say, that was an enormous adjustment.
		(m) I think you only realize that when you are retired. When you don’t have stress with work anymore. Here you can always create an inward peace in yourself and say: “So, we will do this or that…” When we go to work, we have a greater stress factor than when we have more peace.
	SC iii: Retirement (-)	(w) I don’t have so much work anymore. But I still think about my work every day. And I think, “Man, if only you could.” And that makes me a little sick; I was in a hole. That is my story.
		(m) The problem is to bring structure into the day again. That is the big problem.
T III: Differences in women/men	SC i: In general	(w) Well, he [*the husband*] would be helpless with certain things, and with other things I would be helpless. We compliment each other.
		(w) Ladies have other areas of interest than men [*interests of women mentioned here were knitting or sewing, while bowling or rifle club were mentioned as typical interests of men*].
		(m) I don’t know whether that is gender specific. For the most part, yes, but dependent on the type of person. There are strong willed persons on the masculine and the feminine side, and this is characterized in various ways.
	SC ii: Characteristic traits	(w) It is more dramatic. Men are … they think. They are; what should I say? Yes, they are simply pragmatic, more rational.
		(w) That is why they deal with a lot of things inside themselves. They don’t talk about their problems.
		(m) I think, ladies are more ready to accept help than men.
	SC iii: Behavior	(w) He [*the husband*] sooner goes to a doctor for help than I. The complete opposite again.
		(w) Or it takes longer before they go for help … and then, well, when it doesn’t work at all.
		(m) My wife wants to decide very fast. And as soon as she has decided, she gets going. Then she questions everything again.
T IV: Resources/support	SC i: Social environment	(w) If the family does not at least try to accept it at some point, then it is not only difficult, but extremely difficult. And then I think you have great difficulties. You need an anchor, a point where you know you can … Because otherwise everything will explode. That’s what I would say now. Otherwise, if you have someone, and even if you simply dump everything on this person, that helps me.
		(m) My wife has been in psychological treatment for 30 years. And when I was at the point of wanting to lose weight and do other things, I got a lot of help from her. She supported me a lot. And we have had a lot of discussions about it, why we are doing that. I understand that. She gave me a lot of help so I could accomplish my goals.
	SC ii: Healthcare system	(w) But it is always the outer influences and the things around what you experience. Sometimes I think, if I go to my psychotherapist, I always really look forward to that, not only because my psychotherapist is a great therapist but also a wonderful person. And it all has to fit. I really look forward to that. And then I think, oh, what are you going to ask her today? What was it like in the last five or six weeks? What kind of problems did you have? My husband said to me, “Well, tell me. You are 65 years old and you still need input from others on how you need to act?” I say, “Yes, if you have had burnout, then you have collapsed and fallen in a deep depression. And you need someone who can help you out. And if you can’t do it, you need help from somewhere else.” Professional help.
		(m) My general practitioner said it to me. And I was very thankful to him. And then my insurance, which helps me, gave me the offer for a preventative examination. It really helped me that they gave me this information.
	SC iii: Psychological resources	(w) That’s the way it is, that positive attitudes help you to get more power and energy, because you have to.
		(m) (...) because I have a lot of stress at work. And when I was home, I decided to set goals. What do you want to do today? What do you want to do tomorrow? What can you accomplish in this week? Do this and that today. And when I have accomplished that in the evening, I was satisfied and looked for something new to do in the next days. And this calmed me.
	SC iv: Social factors	(w) Multigenerational House
		(w) Home Group
		(m) I think that men and women should work in a community. They should, for example, be active in, above all, sports and rehabilitative sports in a group. Or like we do it: We belong to a choir, my wife and I. These social events give us a lot and open us up. And we can talk, take and give suggestions. Yes, this is very important to communicate with your surroundings.
T V:Barriers	SC i: Social conditions	(w) There is nothing where you can say, “Now give me some advice. Or now, what help can I get?” You are the one who has to see how you deal with these things…
		(m) A lot of group dynamics is involved. “Come, let’s have a smoke together.” And such things. You have to try to avoid such things.
	SC ii: Personal factors	(w) I want to comment on that: for me, this healthy lifestyle with sports, etc., is not possible anymore. We have nothing available for that, or …. So it simply doesn’t work, because I don’t have the energy for that any more. And it all doesn’t work, not sports or …
		(m) It is really very hard for me to say no. And in one day I can overextend myself so much that for the next three weeks I am so overburdened that I can hardly come to rest.
	SC iii: Health care	(w) And unfortunately I have gotten not so much help from our whole health care system. The second treatment at a health resort was denied. They didn’t believe that I had problems with pain. Retirement was denied. I had tried to get another extension at the court and another appointment. It was all denied. And then I had to see how I could deal with that. That was a very big psychological burden.
		(m) And I find that not good, because that is our health care system. It is oriented toward money and not people. And the insurance companies want to earn money, I think. And then, when you are not at death’s doorstep, you won’t get the treatment at a health resort. And then I got the treatment at a health resort. But I had to contribute a number of things to it. I found that negative concerning the health insurance companies.
	SC iv: Infrastructure	(w) There are specific places in the city to go to get help
		(w) But you have your social aspects when getting groceries. You see a familiar face and can speak with the person. In a city it is all anonymous.
T VI: Needs/requests	SC i: Societal	(w) Social aspects in the city, in the commune in the city—that would be nice, if each citizen had the opportunity for that.
		(m) About general practitioners, I would look at it more, if society and the politicians would deal with it more. I don’t want to say that doctors should be paid better, but that they really get more time to personally deal with someone.
	SC ii: Health care	(w) Somewhere where you can get information, if you have lung disease. What are the opportunities? Where is it …
		(m) Support from the Federal Ministry of Nutrition. For example, there are many things that are not put into practice. Why are there not horrible photos on the packaging, as it is with smokers? There has to be a traffic light on packages of fatty meat, and there the color red, of course, and not green.
	SC iii: Culture	(w) Where is there culture for you that lets you forget everything? Earlier we went to the cinema and then back home. And everything was then the way it should be. You don’t have that anymore.

Notes. T = Theme; SC = Subcategory; w = women; m = men.

## Data Availability

The data presented in this study are available on request from the corresponding author but due to privacy restrictions (i.e., names and locations) only in a redacted version. Due to privacy, the data are not publicly available.
